# A novel arabinose-inducible genetic operation system developed for *Clostridium cellulolyticum*

**DOI:** 10.1186/s13068-015-0214-2

**Published:** 2015-03-04

**Authors:** Jie Zhang, Ya-Jun Liu, Gu-Zhen Cui, Qiu Cui

**Affiliations:** Shandong Provincial Key Laboratory of Energy Genetics, Qingdao Institute of Bioenergy and Bioprocess Technology, Chinese Academy of Sciences, 189, Songling Road, Qingdao, 266101 People’s Republic of China; Key Laboratory of Biofuels, Qingdao Institute of Bioenergy and Bioprocess Technology, Chinese Academy of Sciences, 189, Songling Road, Qingdao, 266101 People’s Republic of China; Qingdao Engineering Laboratory of Single Cell Oil, Qingdao Institute of Bioenergy and Bioprocess Technology, Chinese Academy of Sciences, 189, Songling Road, Qingdao, 266101 People’s Republic of China; University of Chinese Academy of Sciences, Chinese Academy of Sciences, 19, Yuquan Road, Beijing, 100049 People’s Republic of China

**Keywords:** Consolidated bioprocessing, Counterselection marker, Inducible gene expression, Metabolic engineering, Targetron

## Abstract

**Background:**

*Clostridium cellulolyticum* and other cellulolytic *Clostridium* strains are natural producers of lignocellulosic biofuels and chemicals via the consolidated bioprocessing (CBP) route, and systems metabolic engineering is indispensable to meet the cost-efficient demands of industry. Several genetic tools have been developed for *Clostridium* strains, and an efficient and stringent inducible genetic operation system is still required for the precise regulation of the target gene function.

**Results:**

Here, we provide a stringent arabinose-inducible genetic operation (ARAi) system for *C. cellulolyticum*, including an effective gene expression platform with an oxygen-independent fluorescent reporter, a sensitive MazF-based counterselection genetic marker, and a precise gene knock-out method based on an inducible ClosTron system. A novel arabinose-inducible promoter derived from *Clostridium acetobutylicum* is employed in the ARAi system to control the expression of the target gene, and the gene expression can be up-regulated over 800-fold with highly induced stringency. The inducible ClosTron method of the ARAi system decreases the off-target frequency from 100% to 0, which shows the precise gene targeting in *C. cellulolyticum*. The inducible effect of the ARAi system is specific to a universal carbon source L-arabinose, implying that the system could be used widely for clostridial strains with various natural substrates.

**Conclusions:**

The inducible genetic operation system ARAi developed in this study, containing both controllable gene expression and disruption tools, has the highest inducing activity and stringency in *Clostridium* by far. Thus, the ARAi system will greatly support the efficient metabolic engineering of *C. cellulolyticum* and other mesophilic *Clostridium* strains for lignocellulose bioconversion.

**Electronic supplementary material:**

The online version of this article (doi:10.1186/s13068-015-0214-2) contains supplementary material, which is available to authorized users.

## Background

Lignocellulosic biomass is the most abundant and renewable raw material on Earth, and its sustainability and effective cost make it an attractive feedstock of carbon source [1-4]. Because of the complex composition and recalcitrant structure, the application of lignocellulose is difficult [5,6]. Consolidated bioprocessing (CBP) is considered an optimal strategy for lignocellulose conversion because it integrates enzyme production, cellulose hydrolysis, and fermentation in one step to reduce cost and simplify processing [6-8]. *Clostridium cellulolyticum* and many other cellulolytic and solventogenic *Clostridium* species are promising CBP candidates because of their capability to degrade lignocellulose, but systems metabolic engineering of clostridial strains is still necessary to satisfy industrialization [9,10].

Although complex cell wall and anaerobic growth conditions of *Clostridium* make genetic manipulation difficult, a few genetic manipulation methods have been developed recently, including gene disruption methods via either homologous recombination or intron retrohoming mechanism [11-14] and heterologous gene expression methods using replicative or integrative plasmids [15,16]. Inducible gene expression tools are required in the metabolic engineering for *Clostridium* strains because the precise regulation of the target gene function is crucial for either the native pathway engineering or heterologous gene introduction in chassis strains [17,18]. Moreover, several clostridial genetic tools in hand can be improved with an effective inducible gene expression system. For example, toxic gene-derived counterselection markers can be developed in *Clostridium* strains by using an inducible promoter for seamless and successive genome editing [19,20]. ClosTron is a gene targeting method derived from a mesophilic mobile group II intron *Ll.ltrB* [21-23]. It has been extensively used in the gene disruption of *Clostridium* strains [24-28], but its high off-targeting activity affects precise genetic engineering [29]. According to the ribozyme-based DNA integration mechanism of targetron [30], the DNA targeting specificity can be improved by precise management of the expression of intron RNA and intron-encoded protein (IEP) [22,31] using a proper inducible gene expression system.

An ideal inducible gene expression system should have high inducing efficiency, stringency, and specificity to an exogenous inducer innocuous to the host cell, with which the expression level of the target gene as well as the function of the related pathway can be easily regulated theoretically [32]. Various inducible gene expression systems have been constructed, such as the well-known isopropyl-β-d-thiogalactoside (IPTG)/lactose-inducible *lac* system, tetracycline-inducible *tet* system, and arabinose-inducible *ara* system [33-35]. Both *lac* system and *tet* system use a repressor binding to the operator *lacO*/*tetO* in the absence of specific inducers, and the addition of the inducer can activate downstream gene expression [34]. Although relatively high background expression has been observed [36], a typical *lac* system LacI-*lacO* is used to develop the inducible T7 gene expression system, which is widely used in *Escherichia coli* strains for heterologous protein expression [37]. When applied in *Clostridium* strains, the inducing efficiencies of lactose-inducible promoters are relatively low [18,38]. In addition to the lactose-inducible promoter, *tet* system has also been used in the controlled gene expression of non-cellulolytic *Clostridium acetobutylicum*, termed Pcm-2tetO1 [20,39]. Pcm-2tetO1 can up-regulate gene expression by 313-fold with anhydrotetracycline as an inducer, which is the highest inducing efficiency among the reported inducible promoters applied in the genus *Clostridium* [18,20,38-42]. The optimal working condition of Pcm-2tetO1 requires a high inducer dosage, but elevated concentration of anhydrotetracycline shows significant inhibitive effects on cell growth [20]. AraC-*P*_*BAD*_ derived from *E. coli* is the most well-known *ara* inducible system, which uses AraC as either an activator or a repressor to control the expression of downstream genes [33], and has been used in several Gram-negative and Gram-positive bacteria [43-46], but not in *Clostridium* strains so far. Thus, novel inducible gene expression systems with high inducing capability as well as proper inducers are required for the metabolic engineering of *Clostridium* strains.

No inducible promoter has been developed for the cellulolytic species *C. cellulolyticum* by far [39]. It is reported that the metabolism of L-arabinose is regulated by *C. acetobutylicum* at the transcriptional level using an AraR-mediated regulation system [47,48]. In the present study, we develop an arabinose-inducible promoter based on the AraR-regulon of *C. acetobutylicum*. Using this inducible promoter, a novel arabinose-inducible genetic operation system, termed ARAi, is constructed for *C. cellulolyticum*. Our results confirm that the ARAi system can be used to regulate gene expression in *C. cellulolyticum* with high stringency and activity. Furthermore, the ARAi system provides an inducible ClosTron method for precise gene knock-out in *C. cellulolyticum*, which can facilitate the systems metabolic engineering of *C. cellulolyticum* and other *Clostridium* strains for commodity and industry.

## Results

### Construction of the arabinose-inducible expression system

Sensitive regulation of L-arabinose metabolism with AraR as the key repressor has been observed and characterized in *Bacillus subtilis* [49] as well as *C. acetobutylicum* [47,48]. The AraR-regulon members of *C. acetobutylicum* include 11 genes involved in arabinose utilization, transport, and conversion via the phosphate pentose pathway [47], in which the expression of gene *ptk*, encoding a phosphoketolase, showed the most sensitivity to the inducer L-arabinose [47]. Hence, to obtain enhanced induction activity, we selected the promoter of *ptk* and the *araR* regulator expression cassette of *C. acetobutylicum* to construct the ARAi system (Additional file 1). Then, 370-bp from the upstream region of *ptk* was used as the promoter P_*ptk*_ to make sure that the binding site of AraR was covered [47]. The self-regulation of AraR was observed [47], so the predicted native promoter and terminator regions of *araR* were employed in the *araR* expression cassette (Additional file 1).

### Controlled expression of an oxygen-independent green fluorescent protein PpFbFPm in *C. cellulolyticum* using the ARAi system

Although the *araR*-binding sequences in *C. acetobutylicum* and *C. cellulolyticum* were distinct [47], whether the ARAi system could be interfered by the indigenous arabinose-inducible system of *C. cellulolyticum* was not certain. Thus, the availability of ARAi system in *C. cellulolyticum* was tested using an oxygen-independent green fluorescent protein PpFbFPm as the reporter [14]. The plasmid pARA-PpFbFPm including both P_*ptk*_-PpFbFPm and AraR expression cassettes was constructed for ARAi-regulated expression of PpFbFPm. The control plasmid pPTK-PpFbFPm was also constructed for constitutive expression of PpFbFPm without the expression of AraR repressor (Table 1, Additional file 1).

**Table 1:** **Bacterial strains and plasmids used in this study**

**Bacterial strains and plasmids**	**Relevant characteristic**	**Sources**
**Strains**		
*E. coli*		
DH5α	*f80dlacZ*Δ*M15*, Δ(*lacZYA*-*argF*)*U169*, *deoR*, *recA1*, *endA1*, *hsdR17*(*r*_*k*_−, *m*_*k*_+), *phoA*, *supE44*, *l*−, *thi*-*1*, *gyrA96*, *relA1*	Transgen
BL21(DE3)	*ompT gal dcm lon hsdS*_*B*_(*r*_*B*_− *m*_*B*_−) *l* (*DE3* [*lacI lacUV5*-*T7 gene 1 ind1 sam7 nin5*])	Transgen
*C. cellulolyticum*		
H10	ATCC35319, wild type stain	ATCC
H10Δ*pyrF*	Derived from H10, *Ccel*_*0614* disrupted via homologous recombination	[11]
H10::MspI297s	Derived from H10, *mspI* ^−^, *Ccel*_*2866*:: MspI297s	[14]
H10Δ*pyrF*::CipC117a	Derived from H10Δ*pyrF*, *Ccel*_*0728*::CipC117a	[11]
H10Δ*pyrF*Δ*mspI*	Derived from H10Δ*pyrF*, *Ccel*_*2866*::MspI297s	This work
H10Δ*pyrF*Δ*cipC*	Derived from H10Δ*pyrF*, *Ccel*_*0728* ::CipC117a	This work
**Plasmids**		
pMTC6	Mls^R^, Amp^R^, *E. coli*-*C. cellulolyticum* shuttle vector, containing *PpFbFPm*, *thl* promoter, *thl* terminator	[14]
pPTK-PpFbFPm	Derived from pMTC6, containing *ptk* promoter instead of *thl* promoter	
pARA-PpFbFPm	Derived from pMTC6, containing ARAi-*PpFbFPm* cassette	This work
pGusA2-2tetO1	pIMP1 containing Pcm-2tetO1 promoter and *gusA* gene	[20]
pARA-GusA	Derived from pARA-PpFbFPm, containing ARAi-*gusA* cassette	This work
pARA-MazE	Derived from pARA-PpFbFPm, containing T7-*mazE* cassette	This work
pARA-MazE/F	Derived from pARA-MazE, containing ARAi-*mazF* cassette	This work
pSY6-*mspI*	Mls^R^, Amp^R^, *E. coli*-*C. cellulolyticum* shuttle vector, *ptb* promoter, containing *mspI* targetron	[14]
pARA-*mspI*	Derived from pSY6-*mspI*, containing ARAi system instead of *ptb* promoter	This work
pARA-PyrF-*mspI*	Derived from pARA-*mspI*, containing *P*_*fd*_-PyrF expression cassette	This work
pGZ-*cipC*	Mls^R^, Amp^R^, *E. coli*-*C. cellulolyticum* shuttle vector, *ptb* promoter, containing *cipC* targetron	[11]
pARA-*cipC*	Derived from pGZ-*cipC*, containing ARAi system instead of *ptb* promoter	This work
pARA-PyrF-*cipC*	Derived from pARA-*cipC*, containing *P*_*fd*_-PyrF expression cassette	This work

**Figure 1:**
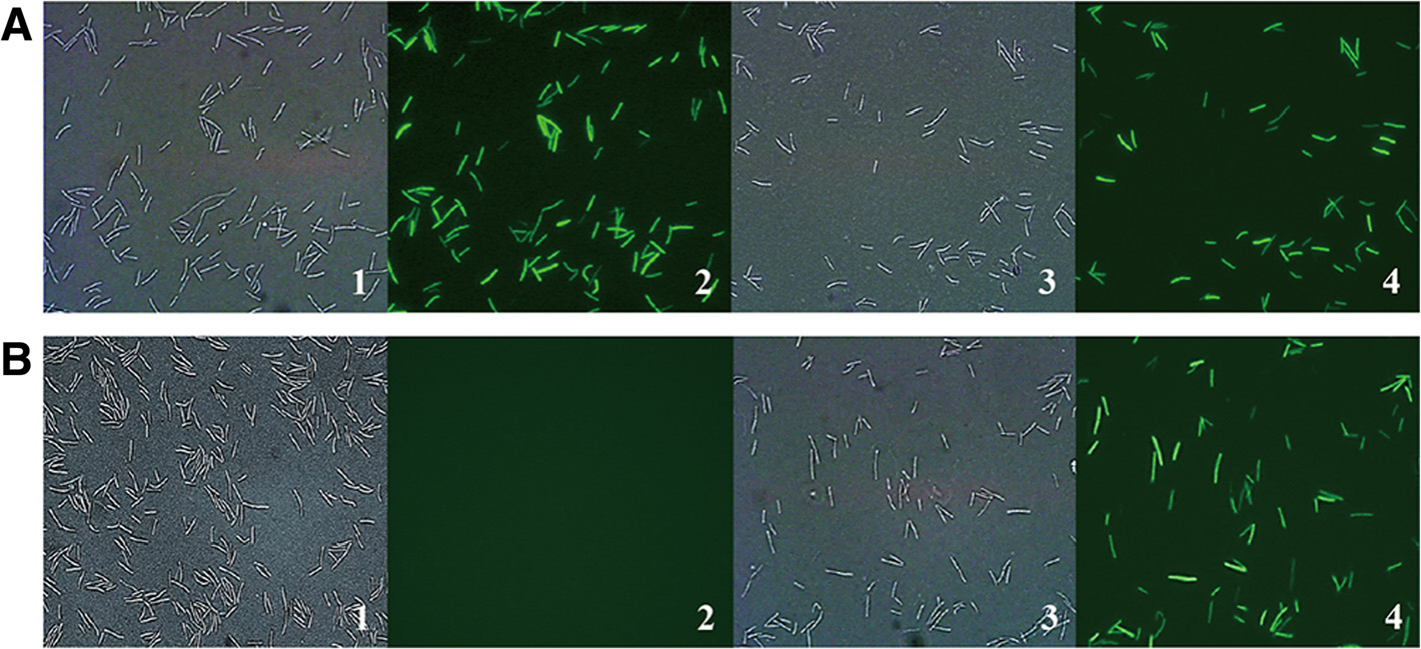
**Confirmation of PpFbFPm expression in** ***C*** **.** ***cellulolyticum*** **H10 by** ***in vivo*** **fluorescent imaging.** *C. cellulolyticum* cells containing pPTK-PpFbFPm **(A)** or pARA-PpFbFPm **(B)** grown at mid-log phase were induced with 10 g/L L-arabinose at 34°C for 2 h or without induction and were used to detect the intracellular fluorescence. 1, 3 corresponds to cell morphology; 2, 4 corresponds to fluorescent imaging; 1, 2 shows cells without induction; 3, 4 shows cells with induction.

*C. cellulolyticum* strains H10::pARA-PpFbFPm and H10::pPTK-PpFbFPm, carrying pPTK-PpFbFPm or pARA-PpFbFPm, respectively, were cultivated with or without addition of L-arabinose, and the intracellular expression of PpFbFPm was investigated by green fluorescence imaging. As shown in Figure 1A, intense green fluorescence was observed in cells of H10::pPTK-PpFbFPm with or without L-arabinose, which confirmed that the *ptk* promoter originating from *C. acetobutylicum* was not recognized and regulated by native AraR in *C. cellulolyticum*. In contrast, H10::pARA-PpFbFPm showed no green fluorescence emission without L-arabinose (Figure 1B), providing a consistent result with previous reports that AraR functioned as a repressor rather than an activator [47]. With addition of L-arabinose, cells of H10::pARA-PpFbFPm showed bright green fluorescence similar to that of H10::pPTK-PpFbFPm, which was indicative of strongly induced expression of PpFbFPm (Figure 1B). These results suggested that the ARAi system was available for controlled expression of target protein in *C. cellulolyticum*. The expression of green fluorescence protein was observed in *E. coli* host cells containing pARA-PpFbFPm whether the inducer was added or not. This indicated the expression leakage in *E. coli*, and suggested that the ARAi system should not be used in *E. coli* strains.

### The efficiency and stringency of ARAi system in *C. cellulolyticum*

The crude enzyme of H10::pARA-GusA was used to determine the efficiency of the ARAi system in *C. cellulolyticum* by β-glucuronidase (GusA) assay using both chromogenic substrate 5-bromo-4-chloro-3-indolyl-β-D-glucuronic acid (X-gluc) and fluorogenic substrate 4-methylumbelliferyl-β-D-glucuronic acid (MUG) [20]. H10::pARA-PpFbFPm was used as a negative control (Table 1).

**Figure 2:**
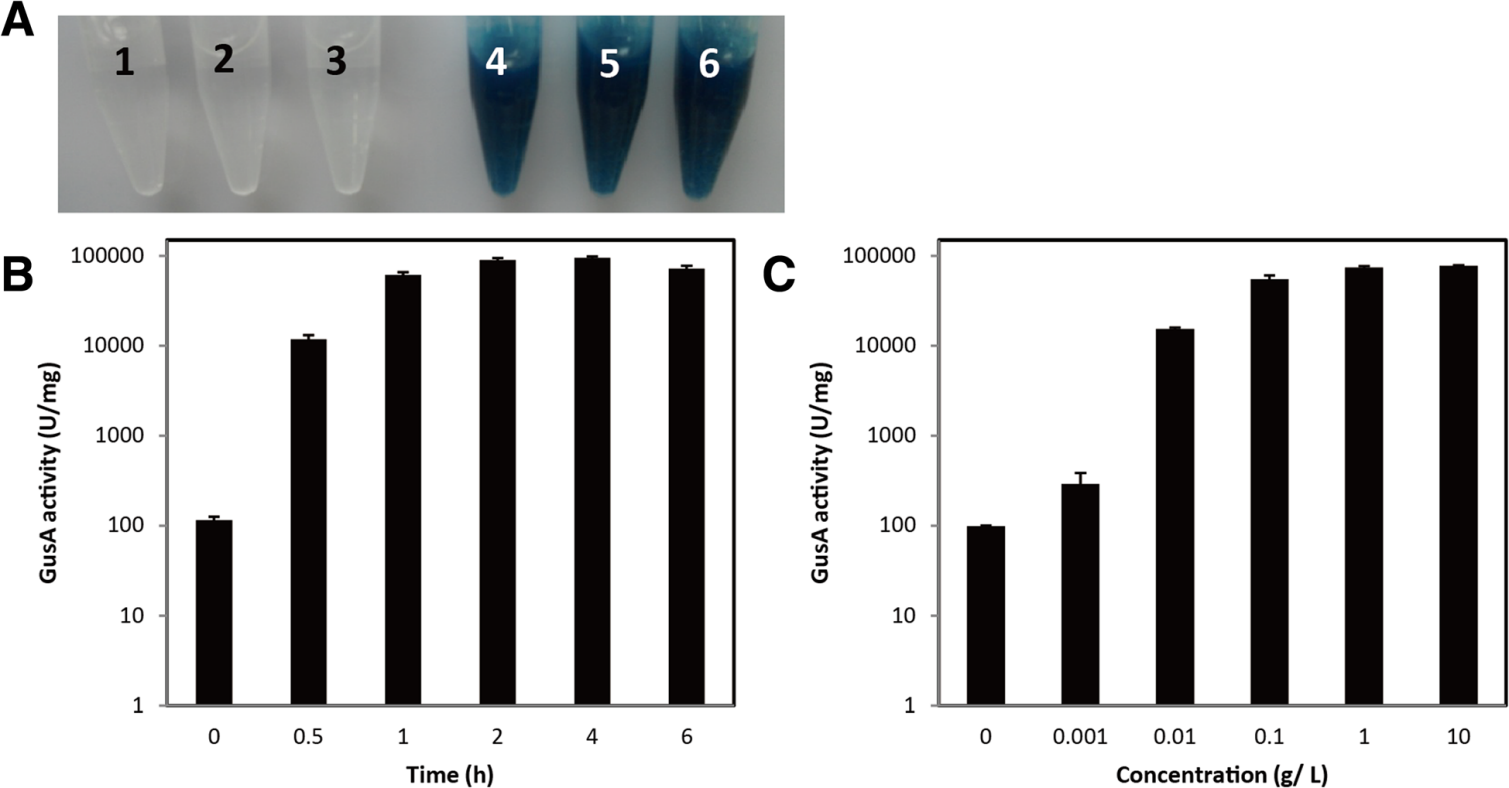
**Investigation of the efficiency and stringency of the ARAi system in** ***C*** **.** ***cellulolyticum*** **H10. (A)** Determination of inducible expression of GusA in H10::pARA-GusA with X-gluc as a substrate. The blue colors of the reaction solutions indicate the induced expression of GusA. The numbered tubes contained: 1, negative control without crude enzyme sample; 2, H10::pARA-PpFbFPm; 3, H10::pARA-GusA without L-arabinose; 4 to 6, H10::pARA-GusA with 0.1, 1 and 10 g/L L-arabinose, respectively. **(B)** Determination of GusA activity during a time course (0 to 6 h) using MUG as a substrate. Then 1 g/L L-arabinose was used as the inducer. **(C)** Determination of the influence of inducer dosage (0 to 10 g/L) on GusA activity using MUG as a substrate. The induction time was 2 h.

With X-gluc as a substrate, the enzymatic reaction mixture of H10::pARA-PpFbFPm showed no change, as well as that of H10::pARA-GusA without induction of L-arabinose, indicating no expression of GusA. However, the crude enzyme of H10::pARA-GusA turned the colorless solution into dark blue when L-arabinose was added (Figure 2A). These results suggested strict and efficient induction activity of ARAi system in *C. cellulolyticum*. When tested with MUG as the substrate, the GusA activity of H10::pARA-GusA was 106.1 ± 8.7 U/mg and was stimulated 100-fold after induction for 0.5 h, indicating that the ARAi system was efficient in *C. cellulolyticum*. H10::pARA-PpFbFPm without L-arabinose induction also showed 26.4 ± 2.8 U/mg activity, which might be a slight expression leakage or the fluorescent background of the reaction system.

To further test the activity of the ARAi system response to induction duration, GusA activity in H10::pARA-GusA was examined during a time course (0 to 6 h) with addition of 1 g/L L-arabinose. GusA activity increased dramatically and reached a maximum level of approximately 9.0 × 10^4^ U/mg after a 2-h induction (Figure 2B). Then, the influence of the inducer dosage on GusA activity was measured by supplementing 0 to 10 g/L L-arabinose as an inducer. The induction time was 2 h. As shown in Figure 2C, GusA activity was significantly stimulated by over 800-fold with increased dosage of the inducer, whereas further addition of L-arabinose from 0.1 g/L up to 10 g/L barely enhanced GusA activity. Thus, the optimum induction condition of ARAi system was 0.1 g/L L-arabinose with 2-h incubation, indicating high inducibility and efficiency of the ARAi system.

### The inducer specificity and inhibition effect on the ARAi system in *C. cellulolyticum*

**Figure 3:**
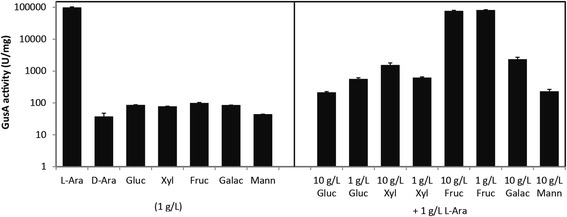
**The inducer specificity and inhibition effect of the ARAi system.** H10::pARA-GusA was first cultivated with cellobiose to mid-log phase, and 1 g/L of various sugars were used to test the inducer specificity of the ARAi system, including L-arabinose (L-Ara), D-arabinose (D-Ara), D-glucose (Gluc), D-xylose (Xyl), D-fructose (Fruc), D-galactose (Galac), and D-mannose (Mann). The inhibition effect of the ARAi system was detected with a mixture of 1 g/L L-arabinose with 1 or 10 g/L of other sugars as inducers. A blank control without inducer was prepared, and the values of all samples were standardized by subtracting the value of the blank control. The induction time was 2 h. Three independent experiments were performed to calculate the average values and standard errors.

In addition to L-arabinose, several sugars including D-arabinose, D-glucose, D-xylose, D-fructose, D-galactose, and D-mannose were used to investigate the inducer specificity of the ARAi system, and the induced GusA activity in H10::pARA-GusA cells was monitored to determine the inducing activity. All selected sugars could be accumulated by *C. cellulolyticum* H10 except D-arabinose. L-arabinose showed over 1000-fold higher inducing activity than the other sugars, which indicated that L-arabinose was the specific inducer of the ARAi system (Figure 3). Surprisingly, the addition of D-xylose, D-glucose, D-galactose, or D-mannose significantly inhibited the induction activity of L-arabinose by approximately 40- to 500-fold, whereas D-fructose showed slight influence (Figure 3).

L-arabinose can be utilized by *C. cellulolyticum* as a carbon source, thus the inducing activity of ARAi may be influenced if the induction period is prolonged. To test the stability of the ARAi system in *C. cellulolyticum*, L-arabinose induction of H10::pARA-GusA was performed for 3 days and both GusA activity and residual L-arabinose and cellobiose in broth were monitored. The results showed that 1 g/L L-arabinose could be completely utilized by H10::pARA-GusA in 12 h, at which time point the GusA activity decreased by 36% (Additional file 2). This indicated that the consumption of L-arabinose affected the inducing activity of the ARAi system. GusA activity was also detected and slightly decreased after 12 h of incubation, which might be explained by the stability of the GusA protein in *C. cellulolyticum* cells or the persistent binding of intracellular L-arabinose with AraR.

**Figure 4:**
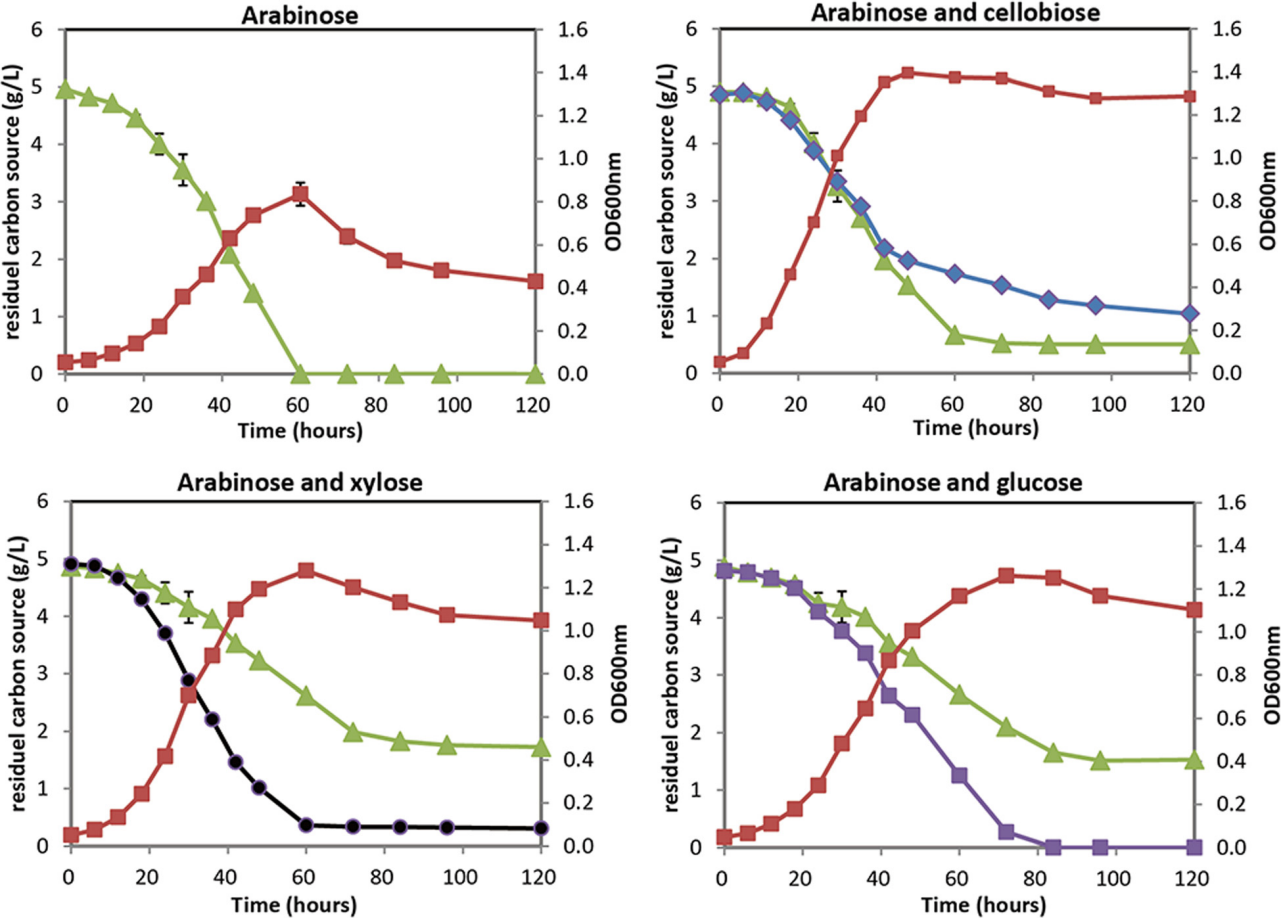
**The growth analysis of** ***C*** **.** ***cellulolyticum*** **H10 using various sugars as carbon sources.** 5 g/L L-arabinose or sugar mixtures (L-arabinose + cellobiose, L-arabinose + D-xylose, or L-arabinose + D-glucose) were used as carbon sources. Cell growth was determined by monitoring the optical density at 600 nm (OD_600nm_, red square). The residual carbon sources in broth were measured by HPLC. Green triangle, L-arabinose; blue diamond, cellobiose; black cycle, xylose; purple square, glucose. Three independent cultivations were used to calculate average values and standard errors.

We further investigated whether the L-arabinose utilization could be affected by the potential carbon catabolize repression (CCR) effect in *C. cellulolyticum* H10 by the addition of cellobiose, D-glucose, or D-xylose with L-arabinose simultaneously. Although no typical CCR diauxie growth was observed, we detected slower utilization of L-arabinose than D-glucose or D-xylose by *C. cellulolyticum* H10 in batch fermentation (Figure 4). L-arabinose (5 g/L) was completely used in 60 h as a single carbon source, whereas the addition of D-glucose or D-xylose decreased the assimilation rate of L-arabinose, and no more utilization was detected after 80 h of cultivation. In contrast, supplementation of cellobiose showed a slight effect on the utilization rate of L-arabinose (Figure 4). The results indicated competitive transport of L-arabinose with other monoses, which might cause the inhibition effect of ARAi system in *C. cellulolyticum*.

### Establishment of a counterselection genetic marker MazF in *C. cellulolyticum* using the ARAi system

Counterselection markers are important genome editing tools for systems metabolic engineering, and one of the prerequisites of developing a toxin-based counterselection marker, such as MazF, is a stringent inducible regulation system to tightly control the toxic gene expression [19]. Using the developed inducible promoter, a counterselection marker MazF was established in the ARAi system for *C. cellulolyticum*.

MazF cleaves mRNA at ACA sequences and is toxic to host cell growth [50], and strictly controlled expression of MazF is essential for the construction of the genetic marker [19,51]. We failed to construct the plasmid pARA-MazF by ligating *mazF* gene directly to the downstream of the ARAi promoter in *E. coli* DH5α, which may be explained by the expression leakage of the ARAi system in *E. coli*. In the MazEF toxin-antitoxin system, MazE acts as the antitoxin and interacts with MazF. Thus, we planned to firstly introduce the expression of *mazE* under the control of a T7 promoter/terminator to protect the host cell from the released MazF [52]. In principle, MazE would only be expressed in strains possessing the T7 RNA polymerase system, such as the *E. coli* BL21(DE3) strain, with the presence of the inducer IPTG, and would not interfere with the function of MazF as a counterselection maker in *C. cellulolyticum*. The plasmid pARA-MazE was successfully constructed by cloning a T7-*mazE* cassette into pARA-PpFbFPm. However, no BL21(DE3) transformants were obtained with or without addition of IPTG when *mazF* was ligated into pARA-MazE. Surprisingly, the target plasmid pARA-MazE/F was successfully created in the *E. coli* DH5α strain containing no T7 RNA polymerase and without IPTG induction (Table 1, Additional file 1), with no clear explanation.

**Figure 5:**
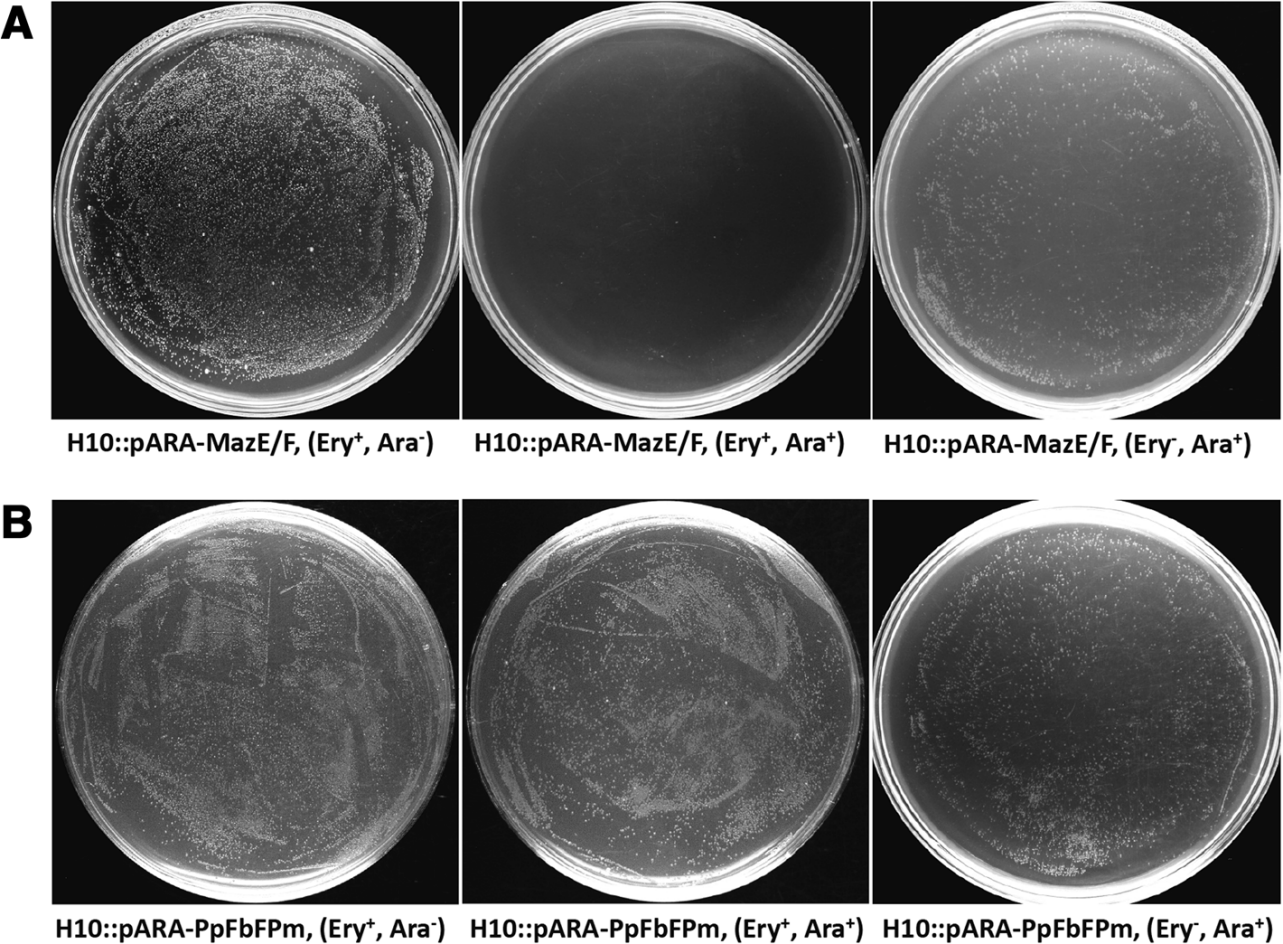
**Availability of the counterselection marker MazF in** ***C*** **.** ***cellulolyticum*** **H10.** Cells of H10::pARA-MazE/F **(A)** and H10::pARA-PpFbFPm **(B)** were plated onto solid GS-2 medium with (superscript +) or without (superscript −) addition of erythromycin (Ery) or L-arabinose (Ara).

pARA-MazE/F was then transformed into *C. cellulolyticum* H10. The obtained strain H10::pARA-MazE/F was plated onto erythromycin-containing solid GS-2 medium with or without addition of L-arabinose to test the availability of the counterselection marker MazF in *C. cellulolyticum*. H10::pARA-PpFbFPm was also tested as a control. In principle, the induced expression of MazF would be lethal to the host cell unless the expression vector was cured. We observed that the growth of control strain H10::pARA-PpFbFPm showed no difference with the presence of either erythromycin (Ery^+^, Ara^−^), L-arabinose (Ery^−^, Ara^+^), or both (Ery^+^, Ara^+^) (Figure 5B). H10::pARA-MazE/F grew regularly on the erythromycin supplemented agar plate without L-arabinose (Ery^+^, Ara^−^), but did not grow when both L-arabinose and erythromycin were added (Ery^+^, Ara^+^) (Figure 5A), suggesting that the expression of toxic MazF was strictly induced by L-arabinose.

In addition, H10::pARA-MazE/F cells grew normally on the agar plate containing only L-arabinose but no erythromycin (Ery^−^, Ara^+^) (Figure 5A). The colonies were randomly selected and inoculated into fresh liquid GS-2 medium with (Ery^+^) or without (Ery^−^) erythromycin, and no growth was observed in Ery^+^ medium. This indicated that without the selection stress of antibiotics, the plasmid pARA-MazE/F was cured due to the induced expression of MazF. Hence, the establishment of the counterselection system pARA-MazE/F not only verified the stringency of ARAi system in *C. cellulolyticum* but also provided an efficient inducible counterselection marker for further seamless genetic manipulation.

### Development of an inducible ClosTron using ARAi system

Because of its high efficiency, ClosTron has been extensively used in the gene disruption of mesophilic *Clostridium* strains [24-26,28], but a deficiency of low target specificity still remains in the targetron methods. We hypothesized that off-target integrations of targetron occurred due to sequence similarity to the desired target site and continuous expression of targetron elements. Therefore, the off-target frequency might decrease if the expression of targetron is controlled using an inducible gene expression system instead of the strong *ptb* (phophotransbutyrylase) promoter [25].

**Figure 6:**
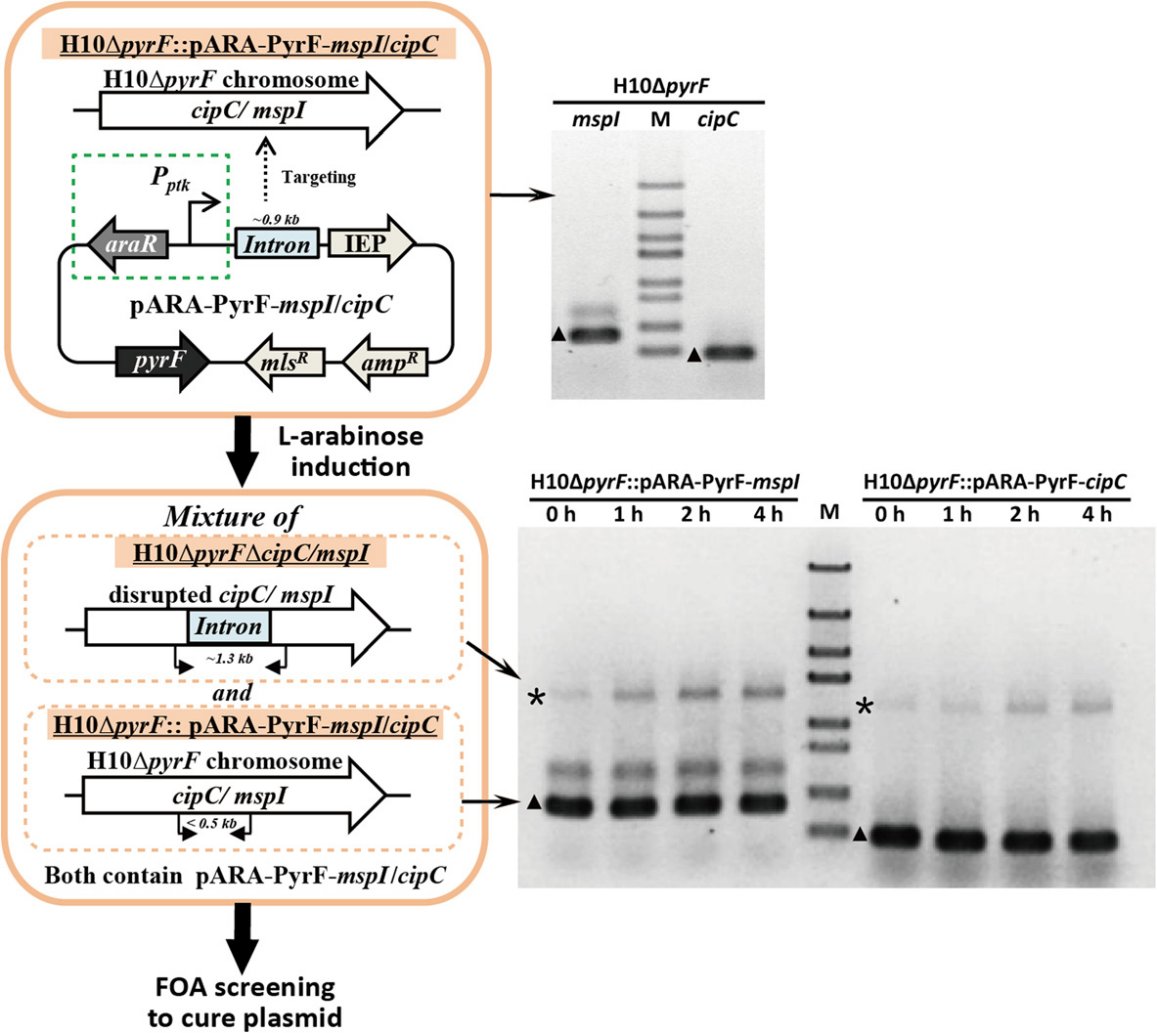
**Schematic representation and PCR confirmation of H10Δ** ***pyrF*** **transformants carrying pARA-PyrF-** ***mspI*** **or pARA-PyrF-** ***cipC*** **before FOA screening.** Green dashed boxes indicated that the developed arabinose-inducible expression system containing a *P*_*ptk*_ promoter and an AraR expression cassette (Additional file 1). The transformants were inoculated and cultivated in antibiotic-free medium until mid-log phase, and 1 g/L L-arabinose was added for induction for 0, 1, 2, and 4 h. Primer sets Ccel2866-F/R and Ccel0728-F/R were used for PCR flanking the intron target sites for gene *mspI* and *cipC*, respectively. The asterisks indicate the PCR products of mutants containing the 0.9-kb intron sequence, and the bands of 300 to 400 bp marked by triangles indicate the PCR products of host strain H10Δ*pyrF*. M, DNA marker (from top to bottom, 5000, 3000, 2000, 1500, 1000, 800, 500, and 300 bp).

To verify this hypothesis, we modified two reported targetron plasmids pSY6-*mspI* [14] and pGZ-PyrF-*cipC* [11] using the developed arabinose-inducible promoter, and obtained pARA-PyrF-*mspI* and pARA-PyrF-*cipC* targeting *mspI* and *cipC* genes, respectively (Table 1, Additional file 1). The new targetron plasmids were transformed into the chassis strain H10Δ*pyrF*, and the desired mutants H10Δ*pyrF*Δ*mspI* and H10Δ*pyrF*Δ*cipC* were obtained following a two-step procedure as described [11], except that, in the first step, transformants were inoculated and cultivated in antibiotic-free medium and L-arabinose induction was performed for controlled expression of intron RNA and IEP. The effect of L-arabinose induction was investigated by PCR before 5-fluoroorotic acid (FOA) screening. The PCR results of transformants carrying pARA-PyrF-*mspI* or pARA-PyrF-*cipC* showed both bands with or without a full-length (0.9 kb) intron sequence (Figure 6), indicating that the colonies were a mixture of host strain H10Δ*pyrF* and mutant strain H10Δ*pyrF*Δ*cipC* or H10Δ*pyrF*Δ*mspI* before plasmid curing (Figure 6). The bands indicating intron insertion at the desired sites became more apparent with longer induction time, which suggested that targetron expression was regulated by the ARAi system, and a novel inducible ClosTron was developed.

The FOA screening was performed to cure targetron plasmids for transformants of pARA-PyrF-*mspI* or pARA-PyrF-*cipC* after 0, 2, or 4 h of L-arabinose induction (Figure 6). Forty-eight or 96 colonies of each transformant were randomly chosen for colony PCR screening, and those colonies with a single band indicating the intron insertion were identified as mutants (Additional file 3). Two to six verified mutants were analyzed using southern hybridization to investigate the off-target frequency of the inducible ClosTron system. The mutants containing single intron insertion were determined as target mutants with no off-target integration, and the number of target mutants was counted to calculate the target specificity by dividing the number of all tested mutants. Both *C. cellulolyticum* mutants H10::MspI297s and H10Δ*pyrF*::CipC117a (Table 1) constructed following the reported ClosTron method [11,14] contained three off-targeted intron insertions in the genomic DNA (Additional file 4). H10Δ*pyrF*Δ*mspI* and H10Δ*pyrF*Δ*cipC* were constructed using the inducible ClosTron. No off-target integration was observed in H10Δ*pyrF*Δ*mspI*, and only one of H10Δ*pyrF*Δ*cipC* mutants showed an additional intron insertion after the arabinose induction for 4 h (Additional file 4). This result indicated that high target specificity could be obtained using the inducible ClosTron.

## Discussion

Controllable regulation of gene expression is essential for either native pathway engineering or heterologous function introduction. Recognizing the importance of inducible gene expression system in metabolic engineering toward the overproduction of certain bioproducts, various inducible promoters have been developed using chemicals, light, or radiation as inducers [32], including the widely used inducible T7 expression system based on the *lac* promoter [33]. This inducible system requires the simultaneous regulation of the expression of T7 RNA polymerase [37], and its expression leakage and cost-inefficient inducer IPTG make it not suitable for difficult microorganisms such as *Clostridium* strains [36]. In this study, we provide a novel arabinose-inducible system ARAi with high inducing stringency and efficiency, and a specific inducible effect to L-arabinose.

L-arabinose is a universal carbon source, and has been used as an efficient inducer in the AraC-*P*_*BAD*_ system derived from *E. coli* [33]. The promoter of AraC-*P*_*BAD*_ system could be repressed by supplementation of glucose because of the CCR effect [33]. For the arabinose-inducible system ARAi developed in this study, the addition of L-arabinose was able to ‘turn on’ the gene expression. The addition of some other monoses can ‘turn down’ the inducing activity in *C. cellulolyticum* (Figure 4), which may benefit the large range of expression regulation. Thus, the ARAi system can be used to flexibly regulate the function of endogenous or heterologous gene for metabolic engineering. In addition, the gene expression can be up-regulated over 800-fold using the ARAi system, which has the highest inducing activity in *Clostridium* so far according to our knowledge. The low inducer dosage will contribute to cost reduction for industrialization. Whereas the influence of other sugars in the medium on the inductive efficiency and the accumulation of L-arabinose by the host cell should also be considered in further applications of the ARAi system.

Counterselection markers are required for efficient chromosomal gene deletion and integration [19], especially in those strains whose genetic manipulations rely on replicative plasmid owing to the low transformation efficiency, such as *C. cellulolyticum*. Two types of counterselection markers are mainly used in *Clostridium* strains. The first type relies on the creation of auxotrophic chassis, such as *pyrF*-disrupted auxotrophic mutant for uracil, and the chassis strains are usually constructed by homologous recombination [11,53]. The second type requires the strict control of toxin expression (for example, *codA* and *mazF*) and the absence of relative antitoxins (for example, *upp* and *mazE*, respectively) [19,20,54]. Benefiting from the high efficiency and stringency of the ARAi system, we successfully constructed an MazF-based counterselection marker. This inducible genetic marker provided the same function of promoting the curing of targetron plasmid (Figure 5) as the reported *pyrF*-based screening system [11], and could be used to achieve seamless genome editing via homologous recombination for systems metabolic engineering of *C. cellulolyticum* and other mesophilic *Clostridium* strains [19].

ClosTron is a popular and convenient tool for gene inactivation, and it has been modified previously for high targeting efficiency. For example, Jia et al. provided a scarless gene deletion approach by combining homologous recombination with ClosTron [55]. Cui et al. disrupted the MspI encoding gene in *C. cellulolyticum* to construct a cell chassis that requires no methylation of heterologous DNA [14], and further developed a *pyrF*-based assistant system for ClosTron to promote plasmid curing and enable successive gene targeting [11]. However, the low target specificity of ClosTron is still a challenge for researchers, and tedious and ineffective analyses with multiple steps are generally required and reinforced to confirm single intron insertion [29]. Highly active off-targeting has also been observed in a newly developed thermotargetron method for thermophiles [12,56]. We consider the off-targeting of targetron to be mainly caused by the short recognition sequence of intron RNA and the persistent expression of intron RNA and IEP, thus the precisely controlled expression of targetron elements may solve the off-target integration problem in *C. cellulolyticum*. Although a modest decrease in target specificity was observed, the application of the ARAi system dramatically enhanced the target specificity of the resulting inducible ClosTron method, and thus solved the problem of off-target integration.

Although *C. cellulolyticum* and *C. acetobutylicum* have a close phylogenetic relationship, and the ARAi system developed in this work as well as several other genetic elements used in *C. cellulolyticum* are derived from *C. acetobutylicum* [11,14,20,25,28], apparent difference in physiological and biochemical properties are present in these two species. For instance, *C. acetobutylicum* cannot degrade cellulose, whereas *C. cellulolyticum* is a typical cellulolytic microorganism producing cellulosome, a multi-enzyme complex assembled on the cell surface [57]; *C. acetobutylicum* barely uses D-xylose as a carbon source with the presence of D-glucose due to the CCR effect, and prefers to use L-arabinose compared to D-xylose [58], while no apparent diauxie growth pattern reflecting glucose-mediated CCR [59-61] has been observed in *C. cellulolyticum*, and D-xylose can be faster consumed than L-arabinose (Figure 3); *C. acetobutylicum* and *C. cellulolyticum* contain different organizations of gene clusters for D-xylose and L-arabinose utilization pathways as well as the DNA recognition motifs of transcriptional regulators [47,62]. The inducing sensitivity of ARAi system in *C. cellulolyticum* is similar to that of AraR-P_*ptk*_ regulon in *C. acetobutylicum* [47]. Thus, it is feasible to investigate the application of the ARAi system in other mesophilic clostridial strains which uptake L-arabinose into the cell.

## Conclusion

This study provided a stringent arabinose-inducible genetic operation system ARAi to support the metabolic engineering of *C. cellulolyticum* and other *Clostridium* strains. The ARAi system includes an effective gene expression platform with an oxygen-independent fluorescent reporter, a sensitive MazF-based counterselection genetic marker, and a precise gene knock-out method based on an inducible ClosTron system, and its inducible effect is specific to L-arabinose. Using the ARAi system, the gene expression was up-regulated over 800-fold with highly inducing stringency, and the target specificity of ClosTron was greatly improved to support precise gene disruption in *C. cellulolyticum*. We suggest that the ARAi system can be widely used for clostridial strains with various natural substrates, and this system will contribute to the efficient metabolic engineering of *C. cellulolyticum* and other mesophilic *Clostridium* strains for lignocellulose bioconversion.

## Materials and methods

### Bacterial strains and cultivation

The bacterial strains used in this study are listed in Table 1. All *E. coli* strains were grown aerobically at 37°C in Luria-Bertani (LB) medium with shaking at 180 rpm or on solid LB plate with 1.5% agar. Then 100 μg/mL ampicillin was supplemented when necessary. *C. cellulolyticum* strains were routinely cultured anaerobically at 34°C in modified GS-2 medium supplemented with 5.0 g/L cellobiose as a carbon source [14]. Furthermore, 20 μg/mL erythromycin or 500 μg/mL 5-fluoroorotic acid (FOA) dissolved in dimethyl sulfoxide was added when required.

### Plasmid construction

All plasmids and primers used in this study are listed in Table 1 and Additional file 5, respectively. Plasmids containing ARAi system were constructed on the basis of pMTC6, an *E. coli* and *C. cellulolyticum* shuttle vector containing an expression cassette of an oxygen-independent fluorescent protein (PpFbFPm) under the control of a thiolase (*thl*) promoter and terminator [14,63]. The ARAi system consisted of a promoter P_*ptk*_ and a repressor AraR. Then 370 bp from the upstream region of the phosphoketolase (*ptk*) encoding gene in *C. acetobutylicum* (CAC1343), the predicted AraR-binding site [47] was amplified with primer set Pptk-F/R and determined as promoter P_*ptk*_. The AraR-expression cassette, including the AraR encoding gene (CAC1340) and its predicted promoter and terminator regions, was amplified with primer set araR-F/R using *C. acetobutylicum* chromosome DNA as a template. The promoter P_*ptk*_ was ligated into pMTC6 using PstI and MluI sites to replace the *thl* promoter and yield the plasmid pPTK-PpFbFPm. The plasmid pARA-PpFbFPm was further obtained by cloning the AraR cassette into the PstI site of pARA-PpFbFPm for inducible expression of fluorescent protein in *C. cellulolyticum* (Figure 1A). pARA-GusA and pARA-MazE/F were constructed based on pARA-PpFbFPm for GusA and MazF expression, respectively. The *gusA* gene was amplified from plasmid pGusA2-2tetO1 [20] using primer set gusA-F/R and ligated into pARA-PpFbFPm by double digestion with NheI and SalI to take the place of *PpFbFPm* (Figure 1A). To construct pARA-MazE/F, *mazE* and *mazF* genes were cloned from the genomic DNA of *E. coli* BL21(DE3) with primer sets MazE-BL21-F/R and MazF-BL21-F/R and ligated into pARA-PpFbFPm in turn using NarI site and MluI and SalI sites, respectively (Additional file 1).

Targetron plasmids were constructed based on pSY6-*mspI* [14] or pGZ-*cipC* [11]. The entire ARAi system including P_*ptk*_ promoter and AraR cassette was amplified from pARA-PpFbFPm with primer set int-araR/int-Pptk, and cloned into pSY6-*mspI* or pGZ-*cipC* to replace *ptb* promoter using XmaI and XhoI sites to generate pARA-*mspI* or pARA-*cipC*, respectively. Targetron plasmids pARA-PyrF-*mspI* and pARA-PyrF-*cipC* were finally obtained by cloning the *P*_*fd*_-PyrF expression cassette into the XmaI site of pARA-*mspI* and pARA-*cipC*, respectively, as described by [11] (Additional file 1).

### Electroporation of *C. cellulolyticum*

Plasmid methylation with MspI methyltransferase, competent cell preparation, and electroporation of *C. cellulolyticum* were precisely performed as described in previous studies [11,14]. All manipulations were performed under anaerobic conditions. Transformants carrying target plasmids were selected on solid GS-2 medium supplemented with erythromycin.

### Fluorescent microscopy

*In vivo* expression of PpFbFPm was monitored with a BX51TRF fluorescent microscope (Olympus Corporation, Shinjuku-ku, Japan). Five hundred microliters of *C. cellulolyticum* cells grown at mid-log phase were harvested by centrifugation, washed twice and resuspended in 20 μL of distilled water. Two microliters of the resuspended cells were used for imaging of intracellular fluorescence (Ex = 460–490 nm, Em = 520 nm).

### β-Glucuronidase assays

For the β-glucuronidase assay, *C. cellulolyticum* strains were cultivated in 100-mL serum bottles until late-log phase with 5 g/L cellobiose as carbon source. Then, 0–10 g/L various sugars (L-arabinose, D-arabinose, D-xylose, D-fructose, D-galactose, or D-mannose) or sugar combinations (L-arabinose + D-glucose, L-arabinose + D-xylose, L-arabinose + D-fructose, L-arabinose + D-galactose, or L-arabinose + D-mannose) were supplemented as inducers. Inducible expression was sustained for 0 to 6 h at 34°C, and the crude enzyme samples were prepared according to a modified protocol [20]. In detail, 30 mL *C. cellulolyticum* cultures were chilled in an ice-water bath for 20 min, and centrifuged at 5000 × *g* for 10 min at 4°C. The cell pellets were washed twice with 30 mL of ice-cold Tris-EDTA buffer (10 mM Tris–HCl, 1 mM EDTA, pH 8.0) and resuspended in 2 mL GusA buffer (50 mM sodium phosphate, 1 mM EDTA, pH 7.0). Cell lysis was achieved by sonication under following conditions: 30% duty cycle, 3 s sonication with a 3 s pause for 10 min (Scientz-IID, Scientz Biotech Company, Ningbo, China). The resulting lysates were centrifuged at 8000 × *g* for 15 min at 4°C, and the obtained supernatants were used as crude enzyme samples for GusA assays. The total protein concentrations of the enzyme samples were determined by the Bradford method [64].

Both chromogenic substrate 5-bromo-4-chloro-3-indolyl-β-D-glucuronic acid (X-gluc) and fluorogenic substrate 4-methylumbelliferyl-β-D-glucuronic acid (MUG) were used to determine GusA activity. For the X-gluc assay, 20 μL of the enzyme samples were mixed with 1 mL of X-gluc assay buffer (GusA Buffer containing 0.086 mM X-gluc) [65], and incubated at 37°C for 2 h until a blue color was observed. When MUG was used as the substrate, the reaction mixture of 0.2 mL diluted enzyme sample and 1.8 mL MUG assay buffer (GusA Buffer containing 4 mM MUG) pre-warmed at 37°C was loaded into a quartz fluorometer cuvette (light path = 1 cm) after adequate mixing, and the fluorescence intensity was measured using a fluorescence spectrophotometer (F-4600, Hitachi Limited, Tokyo, Japan) at 37°C. The fluorescence kinetic curve was recorded with a time-scan mode (Ex = 365 nm, Em = 455 nm, PMT voltage = 700 V) every 30 s for 10 min. The curve slope value and protein concentration of a sample was used to calculate the GusA activity (U/mg) as described [20].

### Mutant screening and plasmid curing

The disruption of *mspI* and *cipC* genes in *C. cellulolyticum* H10Δ*pyrF* using targetron plasmids was performed according to a modified protocol [11]. Transformants containing plasmid pARA-PyrF-*mspI* or pARA-PyrF-*cipC* (Table 1) were inoculated and cultivated in liquid GS-2 medium without erythromycin for 24 h. Then, the cultures were supplemented with 0 to 10 g/L L-arabinose as an inducer at 34°C for another 0 to 4 h. The resulting cultures were washed twice with fresh GS-2 medium, and then plated on solid GS-2 medium supplemented with FOA for plasmid curing based on the reported *pyrF*-based screening system [11]. The plasmid-cured strains were verified if no growth was observed with the presence of erythromycin. The *mspI*- and *cipC*-disrupted mutants were finally confirmed by colony PCR using primer sets Ccel2866-F/R or Ccel0728-F/R (Additional file 5) and sequencing (Sangon, Shanghai, China).

### Southern hybridization

Southern blotting was performed to identify intron insertion in the genomic DNA of *C. cellulolyticum* mutants as described previously [56]. All positive colonies confirmed by PCR and sequencing were inoculated and cultivated in fresh liquid GS-2 medium until late-log phase. Then 4 mL cultures were centrifuged to obtain cell pellets for *C. cellulolyticum* genomic DNA isolation using a TIANamp Bacteria DNA Extraction Kit (Tiangen Biotech, Beijing, China). Genomic DNA was digested with EcoRI and BamHI at 37°C overnight, purified using a traditional phenol/chloroform extraction and ethanol precipitation method, separated by 0.8% agarose gel electrophoresis, and finally transferred to a Nylon membrane (Hybond-NX, GE Healthcare, Pewaukee, WI, USA). The intron probe was amplified using primer set Probe-F/R (Additional file 5) and labeled with digoxigenin-11-dUTP. Hybridization and immunological detection were performed according to the manufacturer’s instructions (DIG-High Prime DNA Labeling and Detection Starter Kit I; Roche, Basel, Switzerland).

### Fermentation analysis

Batch fermentation of *C. cellulolyticum* strains was performed anaerobically in GS-2 medium at 34°C in 250-mL serum bottles with 100 mL medium containing various sugars (L-arabinose, D-xylose, D-glucose, cellobiose) or sugar combinations as carbon sources. Two microliters of broth was sampled every 6 or 12 h during cultivation to analyze the optical density at 600 nm (OD_600_) using a UV-vis spectrophotometer. The supernatants of the samples were obtained by centrifugation and micro-filtration (0.22-μm pore diameter) and were used to quantify the residual sugars directly using high performance liquid chromatography (HPLC) (Rigol L-3000, Rigol Technologies, Oakwood Village, OH, USA) equipped with an Aminex HPX-87H column (Bio-Rad, Hercules, CA, USA) and a refractive index detector (Agilent 1260 infinity RID; Agilent, Santa Clara, CA, USA). Furthermore, 5 mM H_2_SO_4_ was used as the mobile phase at 55°C with a flow rate of 0.5 mL/min [12].

## Additional files

Additional file 1:
**Schematic representation of the plasmids constructed for the arabinose-inducible genetic operation (ARAi) system.** (A) plasmids constructed for inducible gene expression and the counterselection marker; (B) plasmids constructed for the inducible ClosTron system. All plasmids contained an ampicillin resistance gene (*amp* ^*R*^) and an erythromycin resistance gene (*mls* ^*R*^). Green dashed boxes indicated the inducible promoter system containing a *P*_*ptk*_ promoter and an AraR expression cassette from *C. acetobutylicum*. Additional file 2:
**Investigation of inducing activity of the ARAi system in** ***C*** **.** ***cellulolyticum*** **H10 in company with L-arabinose consumption.** H10::pARA-GusA was firstly cultivated with 5 g/L cellobiose for 24 h and 1 g/L L-arabinose was added to induce the expression of GusA. Interval sampling was performed after induction for 2, 6, 12, 24, 48, and 72 h, and GusA activity (Black bars) and the concentration of residual L-arabinose (Green line) and cellobiose (Blue line) were then determined. Two independent setups were prepared and measured for every time point. Additional file 3:
**PCR confirmation of** ***C*** **.** ***cellulolyticum*** **H10 mutant strains.** The transformants of H10Δ*pyrF* containing pARA-PyrF-*mspI* or pARA-PyrF-*cipC* were cultivated in liquid GS-2 medium without antibiotic and induced with L-arabinose for 4 h. After plasmid curing via FOA screening, 48 colonies of each recombinant strain were selected as templates for PCR with primer sets Ccel2866-F/R or Ccel0728-F/R, respectively. Arrows indicated the PCR products (~1.3 kb) with the full-length intron sequence. The bands of approximately 0.4 or 0.3 kb indicate the PCR products of wild-type strain. For H10ΔpyrF::pARA-PyrF-*mspI*, 3 of 48 colonies were confirmed as mutant H10Δ*pyrF*Δ*mspI* (A); for H10ΔpyrF::pARA-PyrF-*cipC*, 6 of 48 colonies were confirmed as mutant H10Δ*pyrF*Δ*cipC* (B)*.* M, DNA marker (from top to bottom, 5,000, 3,000, 2,000, 1,500, 1,000, 800, 500, and 300 bp). Additional file 4:
**Southern hybridization analysis of** ***C*** **.** ***cellulolyticum H10 mutant strains.*** H10Δ*pyrF* was used as a negative control (NC). The inducible ClosTron was employed to construct H10Δ*pyrF*Δ*mspI* (lane 2-4) and H10Δ*pyrF*Δ*cipC* (lane 6-8). Two to six colonies verified as mutants by colony PCR (Additional file 3) were analyzed by southern blotting. The L-arabinose induction was performed for 0 h (lane 2,6), 2 h (lane 3,7) or 4 h (lane 4,8). H10::MspI297s (lane 1) and H10Δ*pyrF*::CipC117a (lane 5) constructed by the reported ClosTron method [1] were also analyzed in parallel. Black triangles indicate the additional off-target insertions. Additional file 5:
**Primers used in this study.**

## Abbreviations

ARAi: arabinose-inducible genetic operation system
CBP: consolidated bioprocessing
CCR: carbon catabolize repression
FOA: 5-fluoroorotic acid
GusA: β-glucuronidase
HPLC: high performance liquid chromatography
IEP: intron-encoded protein
IPTG: isopropyl-β-d-thiogalactoside
LB: Luria-Bertani
MUG: 4-methylumbelliferyl-β-D-glucuronic acid
OD_600_: optical density at 600 nm
PpFbFPm: oxygen-independent fluorescent protein from *Pseudomonas putida*
*ptk*: phosphoketolase gene
*thl*: thiolase gene
X-gluc: 5-bromo-4-chloro-3-indolyl-β-D-glucuronic acid
