# Supplementary material for: A novel arabinose-inducible genetic operation system developed for Clostridium cellulolyticum
Source: Biotechnol Biofuels. 2015 Mar 4;8:36. doi: 10.1186/s13068-015-0214-2 (PMC4355141; doi:10.1186/s13068-015-0214-2)
Supplement: Additional file 1: — Schematic representation of the plasmids constructed for the arabinose-inducible genetic operation (ARAi) system. (A) plasmids constructed for inducible gene expression and the counterselection marker; (B) plasmids constructed for the inducible ClosTron system. All plasmids contained an ampicillin resistance gene (amp R) and an erythromycin resistance gene (mls R). Green dashed boxes indicated the inducible promoter system containing a P ptk promoter and an AraR expression cassette from C. acetobutylicum. [file 13068_2015_214_MOESM1_ESM.docx]

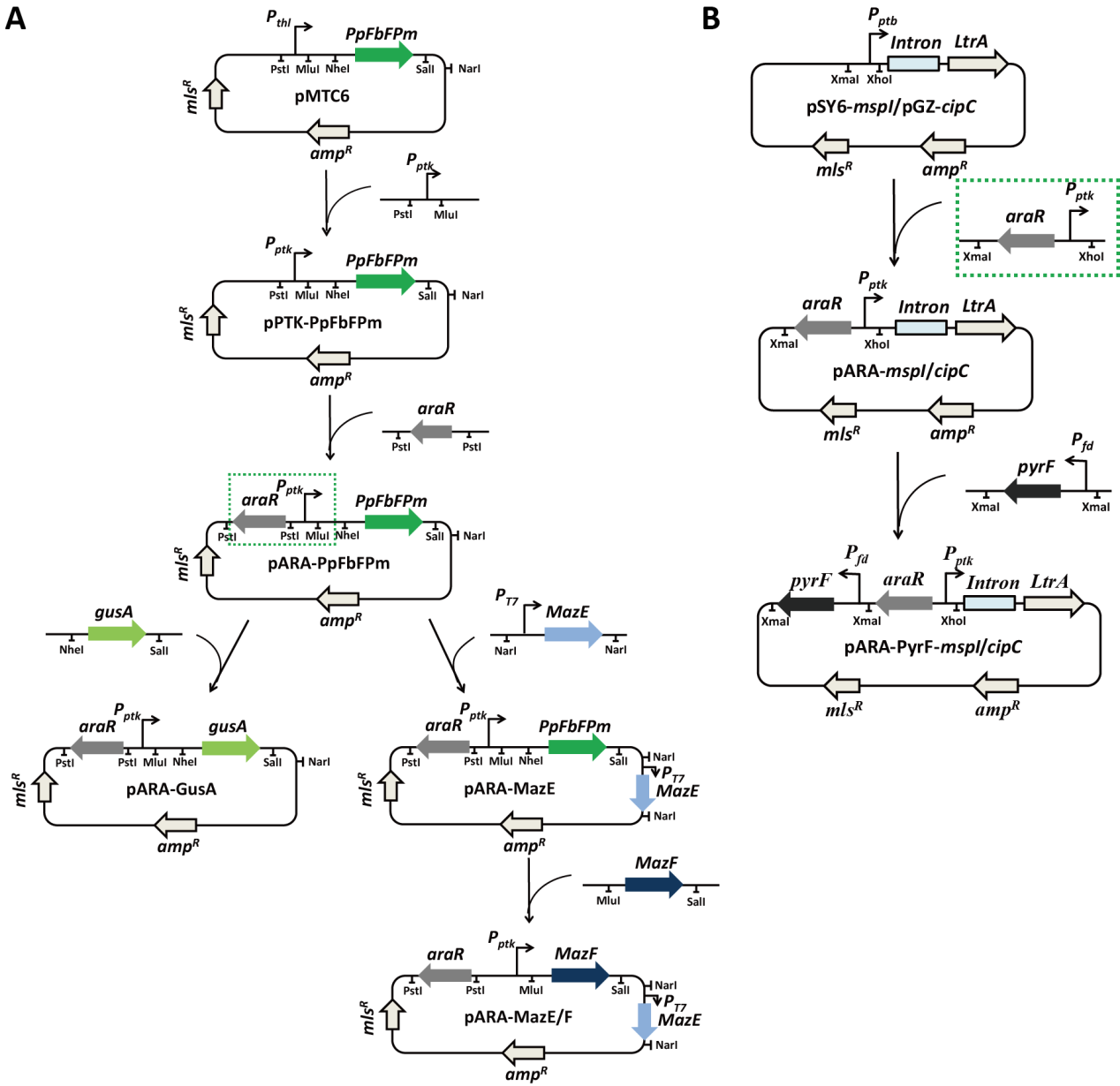


Additional file 1. Schematic representation of the plasmids constructed for the arabinose-inducible genetic operation (ARAi) system.

A, plasmids constructed for inducible gene expression and the counterselection marker; B, plasmids constructed for the inducible ClosTron system. All plasmids contained an ampicillin resistance gene (*amp^R^*) and an erythromycin resistance gene (*mls^R^*). Green dashed boxes indicated the inducible promoter system containing a *P_ptk_* promoter and an AraR expression cassette from *C. acetobutylicum*.
