# Supplementary material for: A novel arabinose-inducible genetic operation system developed for Clostridium cellulolyticum
Source: Biotechnol Biofuels. 2015 Mar 4;8:36. doi: 10.1186/s13068-015-0214-2 (PMC4355141; doi:10.1186/s13068-015-0214-2)
Supplement: Additional file 2: — Investigation of inducing activity of the ARAi system in C . cellulolyticum H10 in company with L-arabinose consumption. H10::pARA-GusA was firstly cultivated with 5 g/L cellobiose for 24 h and 1 g/L L-arabinose was added to induce the expression of GusA. Interval sampling was performed after induction for 2, 6, 12, 24, 48, and 72 h, and GusA activity (Black bars) and the concentration of residual L-arabinose (Green line) and cellobiose (Blue line) were then determined. Two independent setups were prepared and measured for every time point. [file 13068_2015_214_MOESM2_ESM.docx]

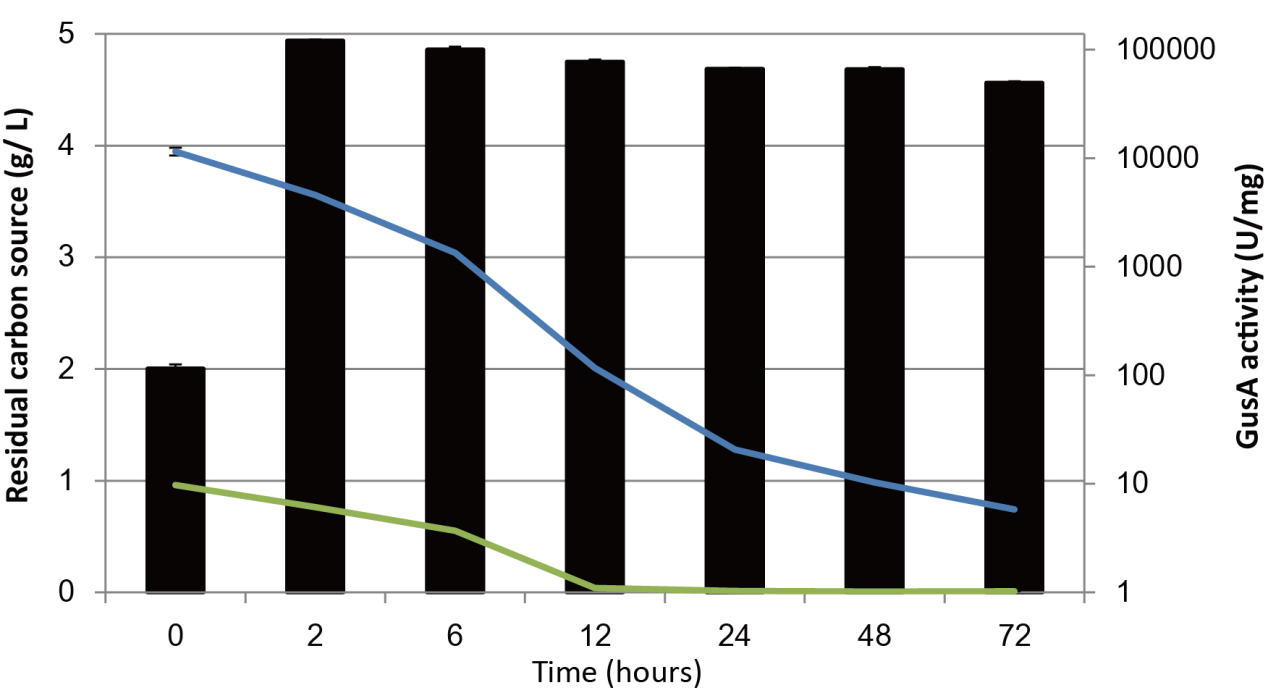


Additional file 2. Investigation of inducing activity of the ARAi system in *C. cellulolyticum* H10 in company with L-arabinose consumption.

H10::pARA-GusA was firstly cultivated with 5 g/L cellobiose for 24 h and 1 g/L L-arabinose was added to induce the expression of GusA. Interval sampling was performed after induction for 2, 6, 12, 24, 48, and 72 h, and GusA activity (Black bars) and the concentration of residual L-arabinose (Green line) and cellobiose (Blue line) were then determined. Two independent setups were prepared and measured for every time point.
