# Supplementary material for: A novel arabinose-inducible genetic operation system developed for Clostridium cellulolyticum
Source: Biotechnol Biofuels. 2015 Mar 4;8:36. doi: 10.1186/s13068-015-0214-2 (PMC4355141; doi:10.1186/s13068-015-0214-2)
Supplement: Additional file 3: — PCR confirmation of C . cellulolyticum H10 mutant strains. The transformants of H10ΔpyrF containing pARA-PyrF-mspI or pARA-PyrF-cipC were cultivated in liquid GS-2 medium without antibiotic and induced with L-arabinose for 4 h. After plasmid curing via FOA screening, 48 colonies of each recombinant strain were selected as templates for PCR with primer sets Ccel2866-F/R or Ccel0728-F/R, respectively. Arrows indicated the PCR products (~1.3 kb) with the full-length intron sequence. The bands of approximately 0.4 or 0.3 kb indicate the PCR products of wild-type strain. For H10ΔpyrF::pARA-PyrF-mspI, 3 of 48 colonies were confirmed as mutant H10ΔpyrFΔmspI (A); for H10ΔpyrF::pARA-PyrF-cipC, 6 of 48 colonies were confirmed as mutant H10ΔpyrFΔcipC (B). M, DNA marker (from top to bottom, 5,000, 3,000, 2,000, 1,500, 1,000, 800, 500, and 300 bp). [file 13068_2015_214_MOESM3_ESM.docx]

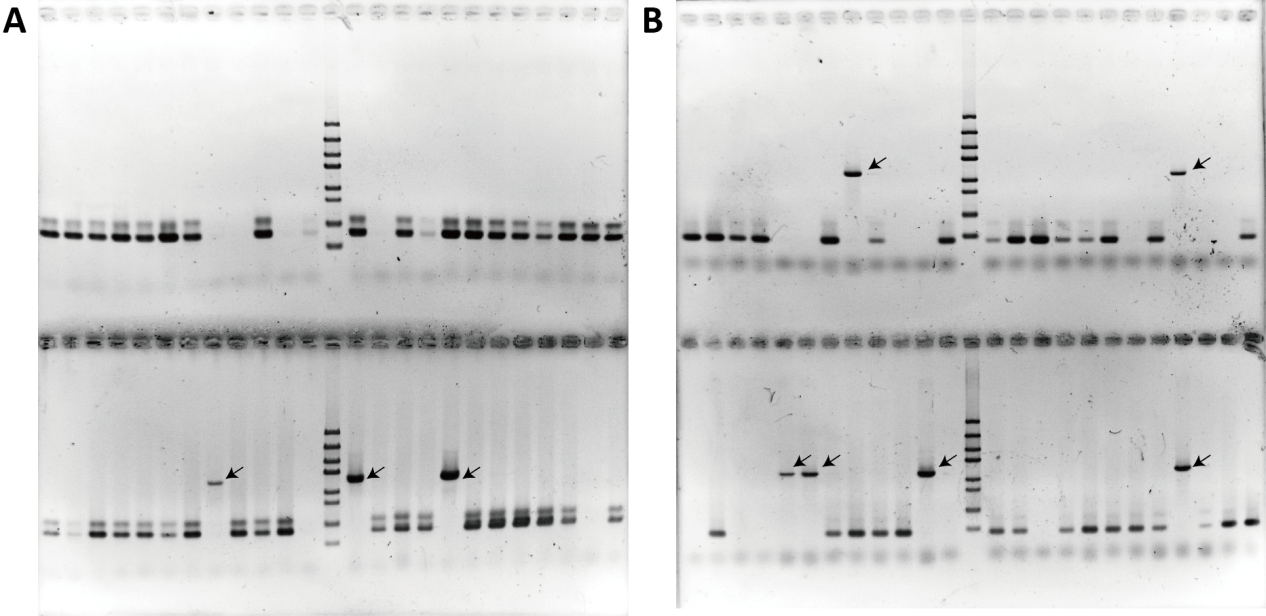


Additional file 3. PCR confirmation of *C. cellulolyticum* H10 mutant strains.

The transformants of H10Δ*pyrF* containing pARA-PyrF-*mspI* or pARA-PyrF-*cipC* were cultivated in liquid GS-2 medium without antibiotic and induced with L-arabinose for 4 h. After plasmid curing via FOA screening, 48 colonies of each recombinant strain were selected as templates for PCR with primer sets Ccel2866-F/R or Ccel0728-F/R, respectively. Arrows indicated the PCR products (~1.3 kb) with the full-length intron sequence. The bands of approximately 0.4 or 0.3 kb indicate the PCR products of wild-type strain. For H10ΔpyrF::pARA-PyrF-*mspI*, 3 of 48 colonies were confirmed as mutant H10Δ*pyrF*Δ*mspI* (A); for H10ΔpyrF::pARA-PyrF-*cipC*, 6 of 48 colonies were confirmed as mutant H10Δ*pyrF*Δ*cipC* (B)*.* M, DNA marker (from top to bottom, 5000, 3000, 2000, 1500, 1000, 800, 500, and 300 bp).
