# Supplementary material for: A novel arabinose-inducible genetic operation system developed for Clostridium cellulolyticum
Source: Biotechnol Biofuels. 2015 Mar 4;8:36. doi: 10.1186/s13068-015-0214-2 (PMC4355141; doi:10.1186/s13068-015-0214-2)
Supplement: Additional file 4: — Southern hybridization analysis of C . cellulolyticum H10 mutant strains. H10ΔpyrF was used as a negative control (NC). The inducible ClosTron was employed to construct H10ΔpyrFΔmspI (lane 2-4) and H10ΔpyrFΔcipC (lane 6-8). Two to six colonies verified as mutants by colony PCR (Additional file 3) were analyzed by southern blotting. The L-arabinose induction was performed for 0 h (lane 2,6), 2 h (lane 3,7) or 4 h (lane 4,8). H10::MspI297s (lane 1) and H10ΔpyrF::CipC117a (lane 5) constructed by the reported ClosTron method [1] were also analyzed in parallel. Black triangles indicate the additional off-target insertions. [file 13068_2015_214_MOESM4_ESM.docx]

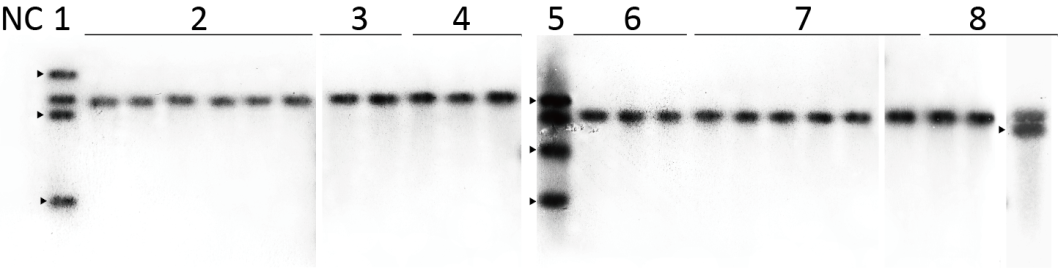


Additional file 4. Southern hybridization analysis of *C. cellulolyticum* H10 mutant strains.

H10Δ*pyrF* was used as a negative control (NC). The inducible ClosTron was employed to construct H10Δ*pyrF*Δ*mspI* (lane 2-4) and H10Δ*pyrF*Δ*cipC* (lane 6-8). Two to six colonies verified as mutants by colony PCR (Additional file 3) were analyzed by southern blotting. The L-arabinose induction was performed for 0 h (lane 2,6), 2 h (lane 3,7) or 4 h (lane 4,8). H10::MspI297s (lane 1) and H10Δ*pyrF*::CipC117a (lane 5) constructed by the reported ClosTron method [[1](#_ENREF_1)] were also analyzed in parallel. Black triangles indicate the additional off-target insertions.

1. Cui GZ, Zhang J, Hong W, Xu C, Feng Y, Cui Q, Liu YJ: **Improvement of ClosTron for successive gene disruption in *Clostridium cellulolyticum* using a *pyrF*-based screening system.** *Appl Microbiol Biotechnol* 2014, **98:**313-323.
