# Supplementary material for: A novel arabinose-inducible genetic operation system developed for Clostridium cellulolyticum
Source: Biotechnol Biofuels. 2015 Mar 4;8:36. doi: 10.1186/s13068-015-0214-2 (PMC4355141; doi:10.1186/s13068-015-0214-2)
Supplement: Additional file 5: — Primers used in this study. [file 13068_2015_214_MOESM5_ESM.docx]

### Additional file 5. Primers used in this study.

| Primers | Sequences (5’-3’)^*^ |
| --- | --- |
| Pptk-F  Pptk-R  araR-F  araR-R  gusA-F  gusA-R  MazE-BL21-F  MazE-BL21-R  MazF-BL21-F  MazF-BL21-R  int-araR  int-Pptk  Ccel2866-F  Ccel2866-R  Ccel0728-F  Ccel0728-R  Probe-F  Probe-R | AAAACTGCAGCAGTTGAGCAAGTTTATGAC (PstI)  AAGCGACGCGTCATAATATTCCTCCTAAATTTAT (MluI)  AAAACTGCAGATATGATCTTCCATAACTTAAC (PstI)  AAAACTGCAGTTATGAAAGCGATTACCTATA (PstI)  CCGCTAGCTAGCATGTTACGTCCTGTAGAAACC (NheI)  TTACGCGTCGACTCATTGTTTGCCTCCCTGCTG (SalI)  ATGGCGCCTAATACGACTCACTATAGGGGAATAATTTTGTTTAACTTTAAGAAGGAGATATACCATGATCCACAGTAGCGTAAAG (NarI)  ATGGCGCCCAAAAAACCCCTCAAGACCCGTTTAGAGGCCCCAAGGGGTTATGCTAGTATCGGCTTACCATTACCAG (NarI)  CGACGCGTATGGTAAGCCGATACGTACCC (MluI)  CCGTCGACCTACCCAATCAGTACGTTAATT (SalI)  TTACCCGGGTTATGAAAGCGATTACCTATAT (XmaI)  CCGCTCGAGAATATTCCTCCTAAATTTATAATC (XhoI)  GGATTAATATAGAAATTAAAGAAGG  CAATACTGGTTTCAGCATCATTAC  CTAACAATGTTGGTGACATTATTAG  CTTTGAGGTGGCATCTATATAGTTG  GAGTCGCAATGTTAATCAGA  AGTCCATAGGCTTAACTTACC |

* Restriction sites are underlined and indicated in following parentheses.
